# Rabies Elimination by 2030: What Challenges Does Iran Face?

**DOI:** 10.18502/ijph.v49i7.3603

**Published:** 2020-07

**Authors:** Hamed EBRAHIMZADEH LEYLABADLO, Hossein BANNAZADEH BAGHI

**Affiliations:** 1. Infectious and Tropical Diseases Research Center, Tabriz University of Medical Sciences, Tabriz, Iran; 2. Student Research Committee, Tabriz University of Medical Sciences, Tabriz, Iran; 3. Immunology Research Center, Tabriz University of Medical Sciences, Tabriz, Iran; 4. Department of Microbiology, Faculty of Medicine, Tabriz University of Medical Sciences, Tabriz, Iran

## Dear Editor-in-Chief

Annually, 300 human cases of rabies are reported in the Middle East with several hundred post-exposure treatments (1). In Iran, on average, nine people die per year due to rabies (2). However, figures provided by Iranian health authorities show that the number of infections and deaths from rabies is largely underestimated in this area. Furthermore, statistics prove that 85% of all people bitten by infected animals live in rural areas and are predominantly male. The group that is most affected by rabies are 11 to 20 yr old. According to official figures, in addition to costs related to rabies community awareness campaigns, 150 billion dollars are spent on the purchase of vaccine and rabies serums per year. Additionally, nearly 700 health centers in 31 provinces of the country (working 24/7) are involved in rabies post-exposure prophylaxis application (3).

Iran still faces many challenges on the road to rabies elimination. Perhaps one of the most difficult issues to overcome is the lack of rabies awareness, particularly in rural areas. Moreover, the fact that dogs and cats are not vaccinated poses a serious health hazard. The last and possibly most important reason is that rabies spreads over borders such as Iraq, Turkey, Pakistan and Afghanistan into Iran very easily. Border provinces such as West Azerbaijan, East Azerbaijan, Kurdistan, Kermanshah, Sistan & Baluchistan, Ilam, Khuzestan and Razavi Khorasan are especially at risk and can be local virus exchange regions between animals from neighboring countries and Iran. This makes rabies an extremely difficult disease to control in and eradicate from Iran. In several countries, including Iran, there is a misconception that killing stray dogs can protect people from the risk of the disease. However, even very large scale killings of thousands of animals in Iran have failed to eradicate rabies in the country. Since dogs are territorial animals, the killing of stray dogs in one area creates a vacuum in this region which invites other stray dogs of other regions to enter the free territory with a surplus of food sources leading to an increase of stray dogs in general.

While dog-mediated rabies is considered to be endemic in Iran, the country is dedicated to the eradication of rabies. This can prove to be problematic however due to neighboring countries, such as Iraq, in which the incidence of human rabies cases exceeds those of Iran drastically. While estimated human rabies cases in 2009 in Iran were 0.02 per million persons it was 0.89 in Iraq (4). The major difference in statistics is believed to be directly linked to unrest and conflict from 2003 onwards, leading to an estimated threefold increase from 2001–2010 (5). Another worrisome fact is the lack of information on rabies from other Middle Eastern countries experiencing unrest such as Syria, their lack of rabies control efforts and the expected transboundary movement of dogs (5).

However spreading awareness about rabies transmission, especially to those in rural areas that work in animal husbandry, can have an immense influence on dog-mediated rabies transmission patterns. Reducing contact to stray animals will protect against rabies transmission, whereas most cases of dog-mediated rabies are often contracted by pet or guard animals not vaccinated (6).

Nevertheless, Iran is experiencing some true development in terms of rabies elimination. Vaccination of stray animals, a practice known to have immense impact on the elimination of dog-mediated rabies, is being implemented in select Iranian cities such as Kiashahr. Additionally, one of the world's highest quality animal rabies vaccines is produced in Iran. Finally, increased cooperation between the Ministry of Health, Veterinary Organizations, the Pasteur Institute and the Department of Environment, means that Iran can eliminate rabies.
